# An Integrated Extraction–Purification Process for Raspberry Leaf Polyphenols and Their In Vitro Activities

**DOI:** 10.3390/molecules28176321

**Published:** 2023-08-29

**Authors:** Jing Yang, Liyang Wu, Tao Wang, Yiqing Zhao, Xiaoqian Zheng, Yongping Liu

**Affiliations:** 1 School of Chemistry and Chemical Engineering, North University of China, Taiyuan 030051, China; wly960816@163.com (L.W.); wt112138@163.com (T.W.); zhaoyiqing217@163.com (Y.Z.); zxq001229@163.com (X.Z.); ypliu321@163.com (Y.L.); 2 Dezhou Industrial Technology Research Institute of North University of China, Dezhou 533034, China; 3 Shanxi Jingxi Biotechnology Co., Ltd., Taiyuan 030051, China

**Keywords:** steam explosion, XDA-6, *Rubus ideaus*, quercetin-3-glucuronide

## Abstract

To improve the utilization value of raspberry leaves, the extraction and purification conditions of phenolic compounds from raspberry leaves were optimized, and the contents of phenolic compounds and the biological activities of extracts were studied. After steam explosion pretreatment at 115 °C for 15 min, raspberry leaf extract with a total phenolic content (TPC) of 136.30~140.51 mg GAE/g was obtained via homogenization and ultrasound-assisted extraction. In addition, the adsorption relationship between raspberry leaf polyphenols and middle polar XDA-6 macroporous resin was best described by the Langmuir model, and tended to be monolayer adsorption. Its adsorption kinetics best resembled the pseudo second-order kinetic model, and it was speculated that this was influenced by multiple factors. According to the optimal integrated extraction–purification process, the TPC of the extracts increased to 738.98 mg GAE/g after one application of purification and 905.27 mg GAE/g after two applications of purification. Moreover, the latter case showed the highest antioxidant activity and α-glucosidase inhibition activity, and the content of the most typical compound, quercetin-3-glucuronide, reached 199.69 mg/g. SE has a double-edged effect, and is more conducive to the release of active substances as a pre-treatment method. This study provides a theoretical basis for the efficient use of raspberry leaves, further improving their medicinal and economic value.

## 1. Introduction

*Rubus idaeus* L. is a perennial shrub of the genus *Rubus* in the Rosacease family, and its fruit is the third most valuable small berry in the world, second only to strawberries and blueberries [1,2]. Although raspberry leaves are a by-product of raspberry production, due to their high content of active ingredients, they are also recorded in the British Pharmacopoeia [3] and in monographs on European herbal medicine [4]. Raspberry shoots have also shown biological activity, and have traditionally been used as medicinal herbs in Eastern Europe [5]. Our previous results showed that raspberry leaf extract with 50% total phenolic content (TPC) had positive effects on gut microbiota during in vitro digestion and fermentation, and reduced the ratio of Firmicutes/Bacteroidetes and the relative abundance of potential pathogens in the feces of all volunteers. The extract also increased the relative abundance of some bacteria, e.g., Enterococcus and Prevotella, that have been shown to have beneficial value in maintaining intestinal health [6]. Moreover, it was shown to have a potential role in HFD-induced body weight control in mice [7]. In addition, the extract of sweet leaf tea (*Rubus Suavissimus*) is also rich in gallic acid, ellagic acid, and rutin, and can effectively alleviate low-grade chronic inflammation, reduce metabolic disorders, and ameliorate the obesity phenotype [8,9]. Among 25 kinds of ellagitannin from unripe raspberry (*Rubus chingii*), Chingiitannin A showed the best inhibitory activities against α-amylase and α-glucosidase [10]. Raspberry leaves are rich in polyphenols, ellagictannins, quercetin, and kaempferol derivatives, and have good antioxidant, anti-inflammatory, and anti-diabetic properties, as well as the ability to improve obesity and intestinal flora balance, thus being of high healthcare value and industrial value [6,10,11,12,13].

With the deepening of the research on raspberry leaves and their extract activity and active ingredients, the required contents of phenolic compounds are becoming increasingly high. How can the yield be improved, the time shortened, and the energy consumption reduced? There are three possible solutions: (1) Optimization of raw material: We found that the leaves of both annual and biennial raspberries harvested in late autumn contained highly active ingredients. Among them, the apical and middle leaves contained more polyphenols, while the old leaves contained more saponins. Their hydrolyzable tannin contents were 1.4–1.6 times those of free phenols, with a maximum of about 300 mg/g, with quercetin-3-glucuronide (Q3GR) being the highest, at about 5–10 mg/g DW [14]. (2) Optimization of the extraction process: As an example, the effects of high-speed homogenization (HG), ultrasonic (US)-assisted extraction, and their combination processes on the extraction of coconut mesocarp polyphenols were compared and analyzed. The TPC of the extract obtained using HG + US reached about 300 mg GAE/g, which is approximately double that of the extract obtained using a single process [15]. Wang et al. [16] also extracted phenolic compounds from the roots and leaves of peony using a combined process. The TPCs from the roots and leaves reached about 80 mg GAE/g and 150 mg GAE/g, respectively, values which were about 50% higher than those obtained using a single process. In addition, steam explosion (SE) technology can be used to convert the saturated vapor pressure of the closed chamber into mechanical energy, so that the tissue gap can be expanded and further exploded. This not only leads to the formation of micropores in the cell wall, so that small molecular substances can be more easily released from the cell, but also depolymerizes the macromolecular polyphenols into small molecules and improves their biological activity [17,18]. For example, Hu et al. [19] reported that, after proper treatment with SE, the TPC of okra seed extract increased from 294.57 to 619.07 mg GAE/100 g, and the ·OH radical scavenging rate increased from 18.78% to 67.34%. It may be that the increased content of phenolic compounds after SE treatment leads to enhanced antioxidant activity [20]. Sui et al. treated tea extracts under different pressures for 3 min, and reported that the DPPH and ·OH scavenging rates reached their highest values when the pressure was 0.2 MPa. However, with a continuous increase in pressure, the scavenging rate decreased [21]. (3) Optimization of the purification process: Macroporous resin is an environmentally friendly styrene derivative with stable physical and chemical properties, a large adsorption capacity, good selectivity, and reusability. It is widely used to remove proteins, polysaccharides, pigments, and lipids from extracts in order to obtain a purified extract with higher bioactivity in theoretical research and industrial production [22]. For example, after purification with D101 macroporous resin, the tannin content of *Coriaria nepalensis* bark increased significantly from 32.5% to 70.6%. Its minimum inhibitory concentrations for *Staphylococcus aureus* and *Escherichia coli* were only 32 μg mL^−1^, values which are lower than those of commercial tannic acid [23].

Therefore, in this work, the apical and middle leaves of annual raspberry (Heritage) were collected in late autumn as the raw material. The extraction process of polyphenols from raspberry leaves was optimized by the combination of US + HG + SE, and then the purification process was optimized with respect to macroporous resin screening, loading sample concentration, volume and pH, elution solvent, and other factors to increase the TPC in the extracts. The effects of the secondary SE on the TPC, antioxidant activity, and anti-amylase and anti-glucosidase activities of the extracts were thoroughly investigated. The results will provide basic data for the large-scale preparation of raspberry leaf extracts, provide raw materials for the downstream application of extracts, and lay a foundation for the comprehensive development of the raspberry industry.

## 2. Results and Discussions

### 2.1. Single Factor Experiment of SE Process

In the field of extracting plant active substances, SE technology plays a role in forming small pores in the cell wall and breaking the hydrogen bonds of compounds [18]. However, it has been reported that as the severity of SE increases, the production of plant phenolic compounds and other bioactive substances usually shows a trend of first increasing and then decreasing. More seriously, excessive SE can even lead to plant carbonization [18,24,25].

**Figure 1:**
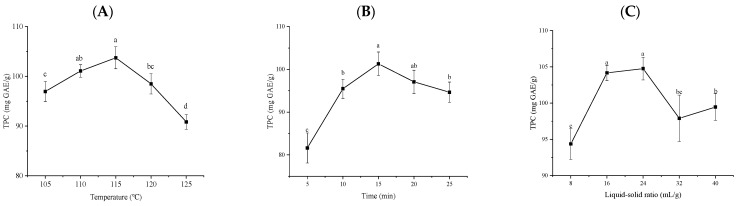
The effect of temperature (**A**), time (**B**), and liquid–solid ratios (**C**) on TPC in SE treatment. Different lowercase letters (a–d) show the significant difference (*p* < 0.05).

Considering plant materials and the safety and energy consumption of the pressure equipment, a set of moderate treatment intensities, i.e., temperature (105–125 °C), time (5–25 min), and liquid–solid ratio (8:1–40:1) were selected as the basic three single factors. As shown in Figure 1A, with the increase in temperature (or pressure), the TPC of the extract solution gradually increased, and reached the maximum value of 103.72 ± 2.21 mg GAE/g at 115 °C (0.168 Mpa). After 115 °C, the TPC gradually decreased. Wan et al. [17] also showed that when the pressure was 0.25–0.75 Mpa for 30 s, the TPC of extracts gradually increased from 1.24 ± 0.07 mg GAE/g DW to 1.77 ± 0.02 mg GAE/g DW. However, at 1.0 Mpa for 30 s, the free phenol content decreased to 1.50 ± 0.06 mg GAE/g DW. Similarly, the TPC of the extract solution increased continuously with the increase in SE treatment time and reached the maximum value of 101.29 ± 2.76 mg GAE/g at 15 min, as seen in Figure 1B. These results indicate that proper vapor pressure can destroy the internal structure of the plant and promote the release of phenolic compounds. Excessive vapor pressure or treatment time can further destroy the structure of all phenolic compounds, resulting in a reduction in TPCs in the later period. Moreover, with the increase in the liquid–solid ratio, the TPC of the extract solution gradually increased and reached the maximum value of 104.76 ± 1.57 mg/g at 24 mL/g, as seen in Figure 1C. A small liquid–solid ratio is not conducive to the full release and dissolution of phenolic acids, while a large ratio affects mass transfer efficiency and wastes solvent [26]. Therefore, a turning point occurred at 24 mL/g.

### 2.2. Response Surface Experiment (RSE) of SE Process

Based on the results of single-factor experiments, the RSE methodology was used to explore the effects of three factors and three levels on the extraction process. Fifteen minutes, 115 °C, and 24 mL/g were designed as the center points for time A, temperature B, and liquid–solid ratio C, respectively, as well as the TPC as the response value Y. The RSE results are shown in Table 1. The fitting equation was

Y = 113.69 + 4.70A + 2.03B + 7.80C − 4.36AB + 1.35AC − 2.55BC − 7.83A^2^ − 14.00B^2^ − 15.31C^2^.

An ANOVA analysis of the results is shown in Table 2. By comparing the F values of the three factors, it can be seen that their impact on the TPC is as follows: F_C_ > F_A_ > F_B_, showing that the effects of the liquid–solid ratio and time on the TPC were greater than that of temperature within the range. Although the temperature (B) had no significant effect on the TPC (*p* > 0.05), the actions of AB and BC (*p* < 0.05) were significantly reciprocal. The R^2^ value of the model was 0.9920, which meant that 99.20% of the variation in response values came from the selected variable, meaning that the model was consistent with the experimental results. This indicator is similar to the results of other RSE studies [16]. In addition, the misfitting term was not significant (*p* = 0.3815 > 0.05), while the Adj R^2^ and the Pred R^2^ were 0.9818 and 0.9299, and their difference was less than 0.2. Therefore, the model could accurately reflect the experimental data and predict the theoretical TPC.

**Figure 2:**
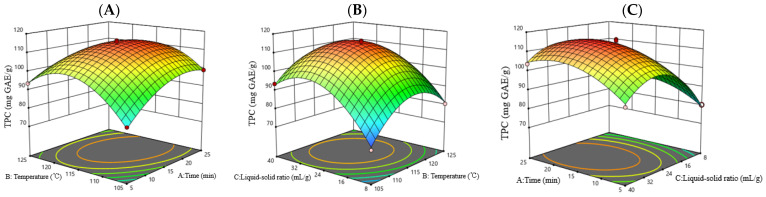
Response surface 3D plots of the interaction effects of three factors on TPC in SE treatment. Temperature and time (**A**), liquid–solid ratio and temperature (**B**), time and liquid–solid ratio (**C**).

According to the above regression equation, response surface 3D plots were used to further explore the interaction between the two factors on the TPC. As shown in Figure 2, under the interaction of the two factors, the TPC showed a trend of first increasing and then decreasing, and the bending degree of the curved surface also reflected the strength of the interaction on the TPC. The reciprocal actions of AB and BC were stronger, while the interaction of AC was relatively weak, which was consistent with the results obtained in Table 2. Overall, a short treatment time at a high temperature or a longer treatment time at a relatively low temperature can be selected.

Finally, the optimal theory extraction process by RSE for the SE-assisted extraction of raspberry leaf polyphenols was 15 min, 113.90 °C, and 28.92 mL/g, and the predicted maximum TPC was 112.09 mg/g. Considering the practical application, the process was slightly adjusted to 15 min, 115 °C, and 29 mL/g. The actual TPC was 113.86 mg/g, which was close to the predicted value.

### 2.3. HG Process and Combination Process

**Figure 3:**
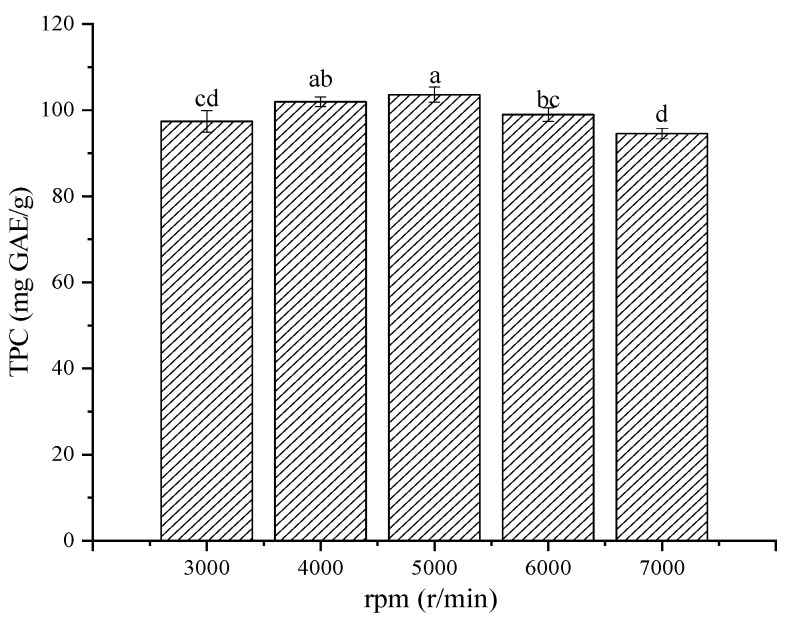
The effect of different speeds in HG treatment. Different lowercase letters (a–d) show the significant difference (*p* < 0.05).

Due to prolonged HG treatment, an unexpected release of polyphenol oxidase may lead to polyphenol degradation [15], and a loss of instrument overheating may occur. Therefore, based on HG for 1 min, the total phenol release within 3000 to 7000 r/min was investigated. As shown in Figure 3, the sample TPC showed a small increase at first and then a downward trend. This may be due to insufficient damage to plant materials at the low HG speed, which limits the effective release of phenolic compounds. However, excessive speed may lead to the destruction of polyphenol compounds caused by polyphenol oxidase [15,27]. Although there was no significant difference in TPC at 4000 and 5000 r/min (>100 mg GAE/g), considering the effect of energy consumption, the optimal HG process was 4000 r/min for 1 min.

**Figure 4:**
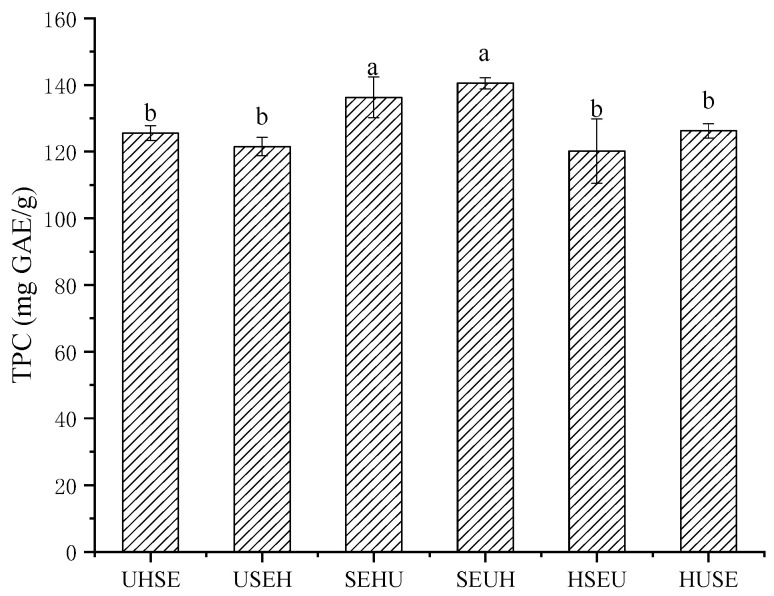
The effect of different combination processes. Different lowercase letters (a, b) show significant difference (*p* < 0.05).

The optimized HG and SE processes in this study, as well as the optimal US process (extraction was performed with 60% ethanol at a liquid–solid ratio of 40:1 mL/g at 120 W for 30 min) [7] were combined into six combinations, as seen in Table 3. Their TPC values are shown in Figure 4 and Table 3. The highest TPCs of the sample by SEHU and SEUH were 136.30 ± 6.08 mg/g and 140.51 ± 1.67 mg GAE/g, respectively (*p* < 0.05), which were higher than those of other processes. These results implied that after SE pre-treatment, the internal structures of plant materials were destroyed. After the subsequent US and HG treatments, the phenolic compounds in the loose internal structure were more easily released and dissolved in the solvent. A similar situation also appeared in a study using SE technology to increase the polyphenol content of mung beans [17]. On the contrary, if SE was used in the second or third step, the TPC was actually reduced. This may be due to the efficient extraction of soluble phenols from leaves at the HG and US steps, which was partially destroyed in the subsequent SE treatment. Thus, SE is a highly destructive condition that requires strict control. SE was more conducive to the release of active substances as a pre-treatment method. The extract obtained via the SEHU or SEUH process was named RLE.

### 2.4. Purification of Phenolic Compounds using Microporous Resin

#### 2.4.1. Preselection of Suitable Microporous Resins and Solution pH

**Figure 5:**
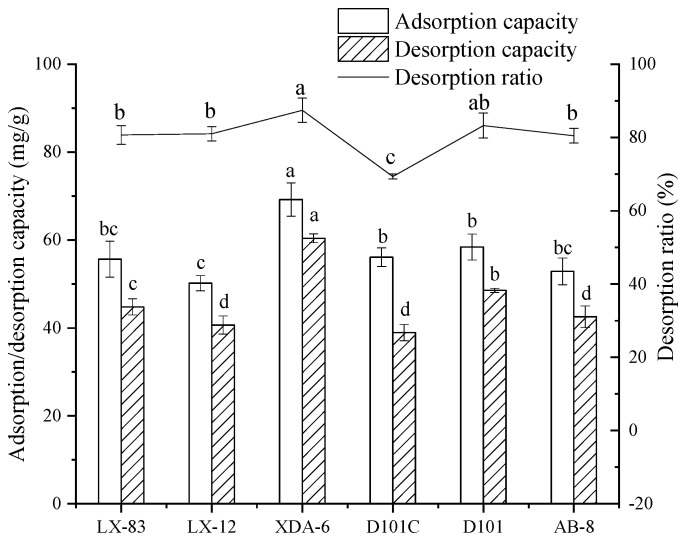
Adsorption/desorption properties of phenolic compounds for six resins. Different lowercase letters (a–d) show the significant difference within the group (*p* < 0.05).

The adsorption capacity, desorption capacity, and desorption ratio of polyphenols in RLE using the six resins were calculated using the TPC (Figure 5). Although the desorption ratios of XDA-6 and D101 were the highest, 87.46% and 83.26%, respectively (*p* > 0.05), the adsorption capacity (58.43 ± 2.95 mg/g) and desorption capacity (48.55 ± 0.47 mg/g) of D101 were much lower than those of XDA-6 (69.22 ± 3.70 mg/g and 60.41 ± 0.98 mg/g). The adsorption/desorption ability of phenolic compounds in macroporous resins is generally related to their polarity and specific surface area [28]. In this study, the specific surface area of XDA-6 was slightly smaller than that of D101, but the polarity of XDA-6 was moderate polarity, which may be more conducive to the adsorption and desorption of phenolic compounds in raspberry leaves. Obviously, XDA-6 was more preferred.

**Figure 6:**
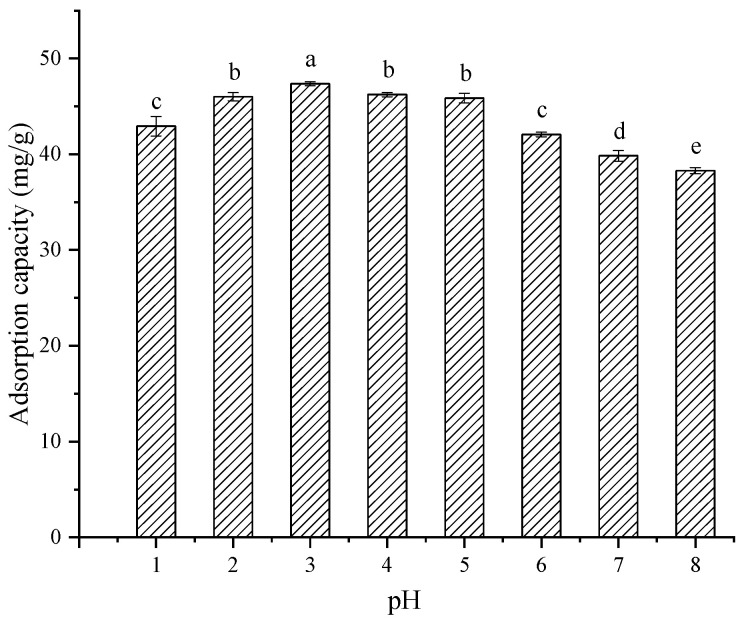
The effect of sample solution pH on the adsorption capacity of XDA-6. Different lowercase letters (a–e) show significant difference (*p* < 0.05).

As shown in Figure 6, the adsorption capacity showed a trend of first increasing slightly and then decreasing. In an acidic condition with pH < 7, the adsorption capacity was >40 mg/g, especially at pH = 3 with a maximum TPC of 47.36 ± 0.21 mg/g, while in an alkali condition with pH = 8, the adsorption capacity was significantly decreased to <40 mg/g. The effect of pH on resin adsorption in the sample is influenced by the degree of ionization of polyphenols. The phenolic compounds are in the form of molecules under acidic conditions, and they are easily adsorbed by the macroporous resin. However, phenolic compounds existing in an ionic form under alkaline conditions are not easily adsorbed by macroporous resins [29]. Therefore, an acidic condition with pH = 3 was selected as one of the subsequent experimental conditions.

#### 2.4.2. Adsorption Isotherm

**Figure 7:**
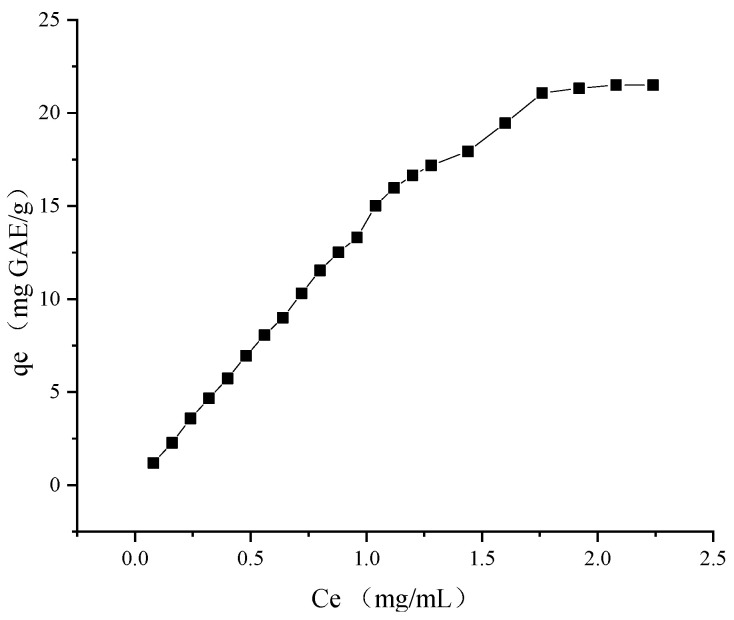
Static adsorption isotherm of XDA-6 macroporous resin.

To understand the adsorption properties of XDA-6 resin on raspberry leaf polyphenols, the effect of different concentration extracts on the adsorption properties was studied at room temperature, and the adsorption isotherm is shown in Figure 7. When the RLE concentration was lower than 1.76 mg GAE/mL, the adsorption capacity of phenolic compounds by XDA-6 resin increased rapidly. After the concentration exceeded 1.76 mg GAE/mL, the adsorption capacity was basically unchanged; hence, the value was selected as the appropriate loading concentration.

The Langmuir adsorption model is used to describe monolayer adsorption, that is, where only one molecule can be adsorbed per adsorption site. Freundlich, an empirical equation, describes that one adsorption site can adsorb more than one molecule [30]. As shown in Table 4, in the Langmuir adsorption model, the q_m_ was 51.93 mg/g, which means that 1 g of XDA-6 macroporous resin can adsorb 51.93 mg of phenolic compounds. The R^2^ = 0.9872 of the Langmuir model was slightly higher than the R^2^ = 0.9721 of the Freundlich model, suggesting that the adsorption mechanism of phenolic compounds in raspberry leaves by XDA-6 was more likely to be monolayer adsorption. It had been reported that after comparing the correlation coefficients of the three models, the adsorption of kale flavonoids by NKA-9 resin also tended to be monolayer adsorption [31]. Moreover, the n value of 1.36 was in the range of 1–10 in the Freundlich model, indicating that XDA-6 resin could easily adsorb phenolic compounds in RLE.

#### 2.4.3. Adsorption Kinetics

**Figure 8:**
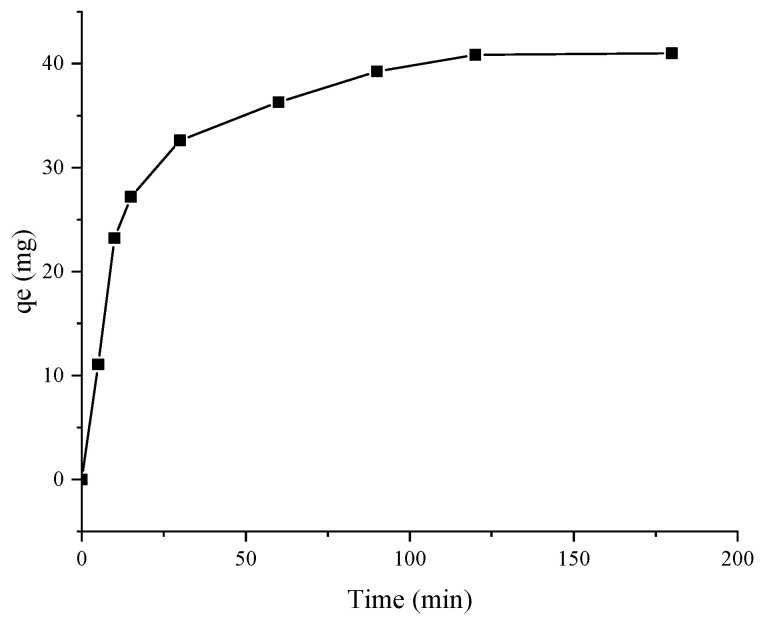
Adsorption kinetics of XDA-6 macroporous resin.

Adsorption kinetics is usually used to analyze the adsorption properties and find the time point of the adsorption equilibrium [32]. As shown in Figure 8, in the initial 60 min, there were many absorption sites in the resin, and there was a large concentration difference between the resin and the extract, which made phenolic compounds more easily enter the resin and become absorbed at room temperature. Therefore, the adsorption capacity of XDA-6 resin to polyphenols increased rapidly. After 60 min, the polyphenol concentration in the resin gradually increased, the adsorption sites were also reduced, and the adsorption capacity of the resin tended to be saturated. The phenolic compounds were not easily absorbed by the resin, so the adsorption gradually reached equilibrium.

The adsorption principle is often predicted using kinetic fitting models. As shown in Table 5, the correlation coefficient R^2^ = 0.9988 of the pseudo-second-order kinetic model was slightly higher than the R^2^ = 0.9460 of the pseudo-first-order kinetic model. Moreover, compared with the actual maximum adsorption capacity of 40.84 mg GAE/g, a maximum adsorption capacity 43.80 mg GAE/g, predicted by the pseudo-second-order model, was closer to the actual value than the 29.13 mg GAE/g result from the pseudo-first-order model. Overall, these results implied that the pseudo-second-order model was more suitable for describing the adsorption behavior of XDA-6 resin on polyphenols from raspberry leaves. Most of the studies on liquid/solid adsorption kinetics show that the pseudo-second-order kinetic model is better than pseudo-first-order models in describing the adsorption process [33]. Generally, this model is assumed to be affected by multiple factors, physical adsorption and chemical adsorption [34]. For example, the benzene rings and hydroxyl groups in the polyphenols of raspberry leaves, adsorbed with the surface and pores of the middle polar resin XDA-6, resulted in a π–π conjugation with the benzene rings of the resin. This adsorption effect may lead to a higher adsorption capacity than that of adlay bran polyphenols (q_e_ < 8 mg/g, [35]).

#### 2.4.4. Effect of Ethanol Concentration on the Desorption Ratio

**Figure 9:**
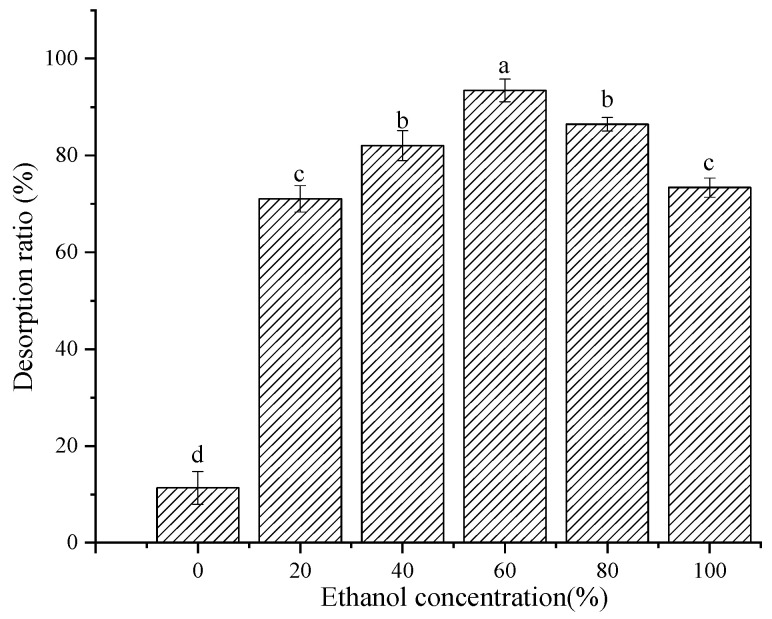
The effect of ethanol concentration on the desorption ratio of XDA-6 macroporous resin. Different lowercase letters (a–d) show significant difference (*p* < 0.05).

The adsorption capacity of the resin is another important index. As shown in Figure 9, the effects of ethanol concentrations of 0, 20%, 40%, 60%, 80%, and 100% on the desorption capacity were investigated. Compared with water (0%), the desorption ratio increased significantly with the increase in ethanol concentration. At 60% ethanol, a maximum desorption ratio of 93.44% was obtained. However, if the ethanol concentration was >60%, then the ratio gradually decreased. This may be because, on the one hand, alcohol-soluble compounds such as chlorophyll, lutein, etc., are desorbed [36]; on the other hand, the solubilities of water-soluble phenolic acids in an alcohol-soluble system are decreased [37]. Therefore, a concentration of 60% ethanol solution was selected as the optimum eluent.

#### 2.4.5. Dynamic Desorption Properties

**Figure 10:**
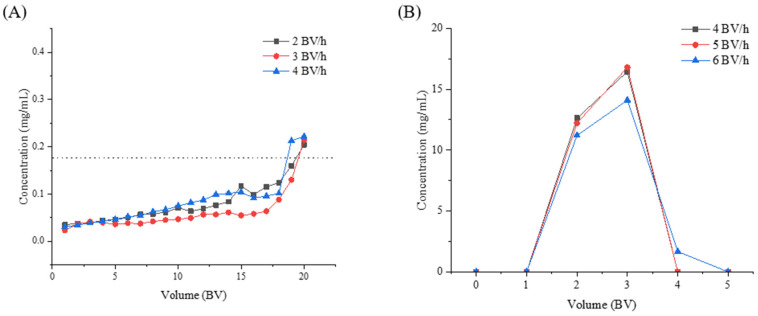
The effect of adsorption (**A**) and desorption (**B**) at different flow rates.

Under different flow rates, the sample solution with pH = 3 and 1.76 mg GAE/mL was used to determine the adsorption leakage curve. When the concentration of phenolic compounds in the outflow solution exceeded 10% of the initial solution, namely > 0.176 mg GAE/mL, this point could be considered as the adsorption leakage point [38]. As shown in Figure 10A, when the flow rate was 2, 3, and 4 BV/h, the corresponding leakage points appeared at the 18th, 19th, and 20th BV, respectively. Considering that the adsorption time was too long at 2 BV/h, and the curve before the adsorption leakage point was smoother at 3 BV/h, a flow rate of 3 BV/h and a loading volume of 19 BV were suitable conditions.

The desorption conditions were investigated using 60% ethanol solution as the desorption solvent under three flow rates of 4 BV/h, 5 BV/h, and 6 BV/h. As shown in Figure 10B, at 6 BV/h, the contact between the eluent solvent and the loaded macroporous resin was insufficient, and the phenolic compounds could not be fully desorbed from the macroporous resin, resulting in the use of more desorption solution. Meanwhile, the release efficiencies at 4 BV/h and 5 BV/h were similar, and their desorptions were sufficient. Based on efficiency priority, 5 BV/h and 3 BV were selected in the subsequent step.

In brief, the optimal adsorption conditions were 1.76 mg/g, pH = 3, and 19 BV at 3 BV/h, and the optimal desorption conditions were 60% ethanol and 3 BV at 5 BV/h. According to the optimal purification process, RLE after one application of purification was named RLP-1, and RLE after two applications of purification was named RLP-2. Their TPCs were 738.98 mg GAE/g and 905.27 mg GAE/g, respectively.

### 2.5. Changes in Phenolic Compounds of Extracts

**Figure 11:**
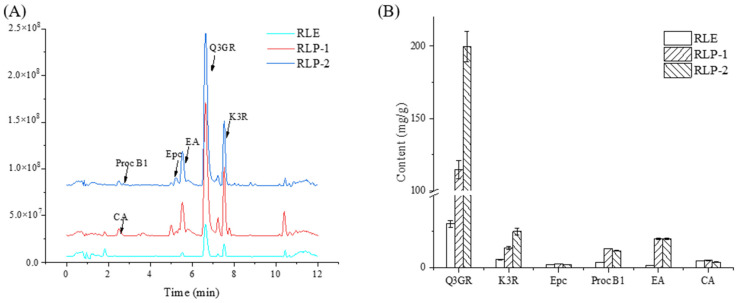
Chromatograms before and after purification (**A**) and the contents of different phenolic compounds (**B**) in the extracts.

Six typical phenolic compounds in RLE, RLP-1, and RLP-2 were compared and analyzed using HPLC-MS. As shown in Figure 11, after purification, the contents of the Q3GR, K3R, proanthocyanidin B1(Proc B1), and ellagic acid (EA) of RLP-1 significantly increased. They were 3.78, 2.37, 3.46, and 12.91 times higher than those of RLE, respectively. Among them, the contents of Q3GR and K3R in the extracts were the highest, and in RLP-2 they were 1.74 and 1.85 times higher than those in RLP-1, respectively, indicating that the effects of the single purification and double purification were significant, especially for phenolic compounds containing glycosides. However, there was no significant change in the contents of epicatechin (Epc) and chlorogenic acid (CA) in RLP-1 and RLP-2, which was different from our previous results. We found that the purification of XDA-6 was beneficial to the enrichment of 11 polyphenols in unripe raspberry extract and raspberry leaves, including Epc and CA [7]. This may be due to the low content of the two compounds after SE pretreatment, meaning that they did not have the advantage of competing with high-content compounds for adsorption sites during the secondary purification stage.

Additionally, these extracts were treated at 115 °C for 10 to 50 min again, and the results showed that only the TPC of RLE showed a small increase in 20 min, while the other treatment groups showed a constant or decreasing trend (Figure S1). SE treatment can cause the depolymerization, oxidation, and thermal degradation of phenolic molecules, thereby changing the molecular structure or forming other compounds. For example, in a study of Sumac fruit coat, 85% quercitrin was converted to quercetin at 200 °C for 5 min [17,39]. A variety of ellagitannin were depolymerized into EA and condensed tannins were depolymerized into catechin, Epc, and Proc B1 after treatment at 121 °C for 30 min [40]. Bound phenolics were hydrolyzed into their free forms and flavonoid glycosides were hydrolyzed and converted into free aglycones via SE [41,42]. Additionally, SE can also disrupt simple phenolic structures until plant carbonization occurs [18]. Here, Epc, EA, and GA in the extracts after SE treatment showed a relative increase, while Q3GR, K3R, Proc B1, and CA showed a relative decrease (Figure S2). This result was consistent with the fact that only EA, catechin, Epc, and Proc B1 in the extract were enriched after 121 °C for 30 min, while Q3GR and K3R were significantly decreased [7,40]. Based on the double-edged effect of SE, it can be selected for treatment in the extraction stage of the raw material or in the post-processing stage of the extract.

### 2.6. Antioxidant and Inhibitory Enzyme Activities of Extracts

The chemical structure and composition of phenolic compounds are very complex, so the antioxidant activity varies greatly. There are many factors that affect the antioxidant properties of phenolic compounds, such as the source of phenolic compounds, chemical structure, molecular weight, purity, and processing technology. The antioxidant activities of RLE, RLP-1, and RPL-2 were evaluated by measuring the scavenging rate of DPPH and ABTS. As shown in Table 6, their IC_50_ values of DPPH scavenging rate were 67.71 μg/mL, 33.99 μg/mL, and 20.63 μg/mL, respectively. Additionally, their ABTS scavenging rates were 14.72 μg/mL, 7.81 μg/mL, and 5.98 μg/mL, indicating that the antioxidant ability was improved after purification, with improvements in TPC. In particular, RLP-2 was closed to the standard Vc level in both methods, demonstrating its potential as an antioxidant candidate.

**Figure 12:**
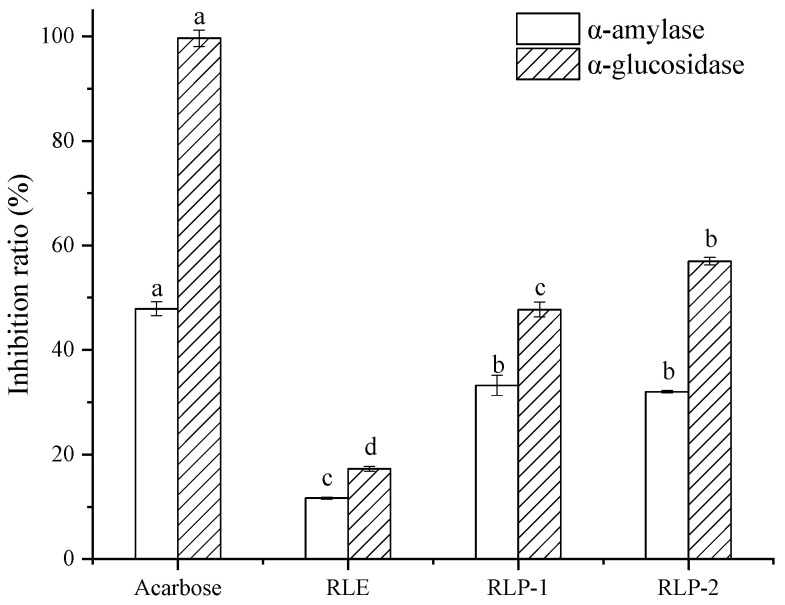
Inhibition rates of α-amylase and α-glucosidase by three extracts. Different lowercase letters (a, b) show the significant difference within the group (*p* < 0.05).

The main role of digestive enzymes is to break down carbohydrates into small units that are taken up by the organism. α-Amylase is responsible for breaking down starch and producing disaccharides in the saliva and pancreas, which are then further broken down into glucose by α-glucosidase in the intestine and absorbed, raising blood glucose levels [43]. Type 2 diabetes mellitus is characterized by an abnormal increase in blood glucose immediately after a meal. The inhibition of α-amylase and α-glucosidase activity can inhibit intestinal glucose absorption and thus reduce blood glucose [44]. Therefore, the inhibition rates of the obtained samples on α-amylase (1 mg/mL) and α-glucosidase (30 μg/mL) at the same concentration were studied, and acarbose was used as a positive control. As shown in Figure 12, the highest inhibition rate on α-amylase was 33.23% for RLP-2 among the three extracts, and that of acarbose was 47.90%. The highest inhibition rates of α-glucosidase for RLP-1 and RLP-2 were 47.73% and 56.99%, respectively, while that of acarbose was 99.65%. Although the extracts had a higher inhibitory rate on α-glucosidase, RLP-2 reached about 70% of the control against α-amylase, while it was less than 60% of the control against α-glucosidase. Hence, RLP-2, including RLP-1 and RLE, may be more sensitive to inhibiting amylase. This provides an enzyme activity basis for the effect of raspberry leaf extract on weight loss and lipid reduction in high-fat-food-induced obese mice. After PAD purification, the leaf extract of *Sauropus androgynus,* 0.61 mg/mL inhibited α-glucosidase slightly more than the acarbose (0.5 mg/mL) [45].

## 3. Materials and Methods

### 3.1. Plant Materials and Reagents

The apical and middle raspberry leaves (*Rubus ideaus* L. cv. Heritage) were collected at the plantation of the North University of China, Taiyuan, Shanxi Province (38°01′71″ N, 112°44′46″ E), in October 2022. The leaves were air-dried naturally and stored at room temperature until further analysis.

MS-grade acetonitrile and methanol were purchased from Thermo Fisher Scientific—CN. Other reagents were purchased from Shanghai Aladdin Biochemical Technology Co., Ltd. and Yuanye Biotechnology Ltd. (Shanghai, China).

### 3.2. Total Phenolic Content (TPC)

The TPC was determined using the Folin phenol method: 100 μL of sample, 300 μL of Folin phenol, 1.5 mL of 7% Na_2_CO_3_, and 3.1 mL of deionized water were added to the centrifuge tube and incubated for 2 h at room temperature and in the dark. The TPC was calculated using A = 1.808C + 0.056, where A is the absorbance of the sample and C is the concentration of the sample, with TPC expressed as mg GAE/g.

### 3.3. The Extraction Process

**Figure 13:**
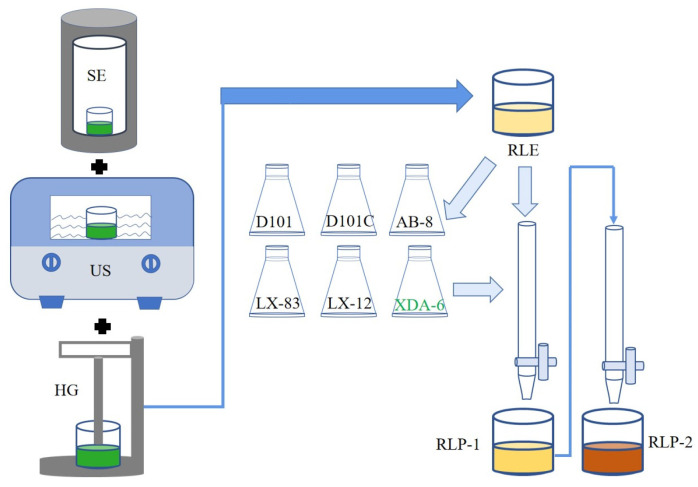
Schematic diagram of the integrated extraction–purification process for raspberry leaf polyphenols in this work.

A schematic diagram of raspberry leaf polyphenols extracted under different conditions is shown in Figure 13.

#### 3.3.1. SE-Assisted Extraction

Raspberry leaf powder (1 g) was passed through a 50 mm sieve and 32 mL deionized water was added and both were mixed together in the flask. Sample solutions were placed in a high-pressure device (BXM-30R, Shanghai Boxun Biotechnology Co., Ltd., Shanghai, China) with a maximum temperature of 125 °C. The effects of temperature, time, and liquid–solid ratio of SE treatment on TPC in the extract were studied in sequence. The single-factor experiment design is displayed in Table 7. The other basic conditions were 110 °C, 10 min, and 32 mL/g.

According to the results of the single-factor experiment, time A, temperature B, and liquid–solid ratio C were selected as the three factors of RSE of SE, and the sample TPC was used as the response value Y. The three levels of each factor with 1, 0, and −1 were designed using the Box–Behnken model in Table 8.

#### 3.3.2. HG-Assisted Extraction

Raspberry leaf powder (1 g) was passed through a 50 mm sieve and 40 mL 60% ethanol solution was added and both were mixed. Sample solutions were homogenized using a high-speed disperser at 3000, 4000, 5000, 6000, and 7000 r/min for 1 min, respectively. The HG condition was provided using a high-speed disperser (XHF-DY, Ningbo Xinzhi Biotechnology Co., Ltd., Ningbo, China) with a maximum speed of 10,000 r/min. After centrifugation (4000 rpm × 5 min), the sample TPC was determined.

#### 3.3.3. Combination Processes

The US condition was provided using a thermostatic water-bath US device (SB-5200 DTD, Ningbo Xinzhi Biotechnology Co., Ltd.) with a maximum power of 360 W. Moreover, the US + SE + HG combinations are displayed in Table 3, and the steps were as follows.

Combination 1:

① HG: Raspberry leaf powder (1 g) and 40 mL/g of 60% ethanol solution under 4000 r/min HG treatment for 1 min, obtaining sample solution 1 (SS1).

② US: SS1 was treated under 120 W US for 30 min, obtaining sample solution 2 (SS2).

③ SE: After the ethanol of SS2 was removed via rotary evaporation, about 15 mL of deionized water was added to obtain 29 mL/g. This was treated under 115 °C for 15 min, obtaining sample solution 3 (SS3). Combination 6: Exchange ① and ②.

Combination 2:

① HG: Raspberry leaf powder (1 g) and 40 mL/g of 60% ethanol solution under 4000 r/min HG treatment for 1 min, obtaining sample solution 1 (SS1).

② SE: After the ethanol of SS1 was removed via rotary evaporation, about 15 mL of deionized water was added to obtain 29 mL/g. This was treated under 115 °C for 15 min, obtaining sample solution 2 (SS2).

③ US: After drying SS2 to 16 mL, 24 mL absolute ethanol was added to reach approximately 40 mL/g of 60% ethanol solution. This was treated using 120 W US treatment for 30 min, obtaining sample solution 3 (SS3). Combination 5: Exchange ① and ③.

Combination 3:

① SE: Raspberry leaf powder (1 g) and 29 mL deionized water under 115℃ for 15 min, obtaining sample solution 1 (SS1).

② HG: After drying SS1 to 16 mL, 24 mL absolute ethanol was added to reach approximately 40 mL/g of 60% ethanol solution. This was treated under 4000 r/min HG treatment for 1 min, obtaining sample solution 2 (SS2).

③ US: SS2 was treated under 120 W US for 30 min, obtaining sample solution 3 (SS3). Combination 4: Exchange ② and ③.

Finally, the TPC of SS3 was determined.

### 3.4. The Purification Process using Macroporous Resin

A schematic diagram of purified raspberry leaf polyphenols is shown in Figure 13.

#### 3.4.1. Macroporous Resin Pretreatment

The macroporous resin was soaked in 95% ethanol overnight, and then washed with deionized water until becoming neutral. Secondly, the resin was soaked in 4% HCl (*v*/*v*) and 4% NaOH (w/v) for 4–6 h in turns, and washed with deionized water until neutralization each time. Finally, it was stored in 95% ethanol solution. The properties of the six macroporous resins are shown in Table 9.

#### 3.4.2. Screening Macroporous Resins

One gram of macroporous resin and 20 mL of extraction solution were added to a 250 mL conical flask. The flask was shaken in an air bath incubator at 25 °C and 150 rpm for 12 h. The adsorption capacity was calculated according to Equation (1). After the solution was removed, the macroporous resin was washed with deionized water, and then 30 mL of 60% ethanol was added. The resin was shaken at 25 °C and 150 rpm for 12 h. The desorption capacity was calculated according to Equation (2), and the desorption ratio was calculated according to Equation (3).

(1) $$q_{a}=\frac{(C_{0}-C_{a})V_{a}}{m}$$

(2) $$q_{b}=\frac{C_{b}V_{b}}{m}$$

(3) $$D\;(\%)=\frac{C_{b}V_{b}}{(C_{0}-C_{a})V_{a}}$$

q_a_ and q_b_ are the equilibrium adsorption capacity and desorption capacity, respectively. C_0_, C_a_, and C_b_ are the TPCs of the initial extract solution, the solution after adsorption, and the desorption solution, respectively. V_a_ and V_b_ are the volumes of extraction solution and desorption solution, respectively. m is the mass of resin and D is the desorption ratio.

#### 3.4.3. The pH of the Extract Solution

The pH of the extract solution was adjusted to 1, 2, 3, 4, 5, 6, 7, and 8 using 4% HCl or 4% NaOH. Other treatments were as used, as shown in Section 3.4.2. Their corresponding adsorption capacities were calculated using their TPC.

#### 3.4.4. Adsorption Isotherm

The TPC of RLE was diluted to 0.32, 0.48, 0.64, 0.80, 0.96, 1.12, 1.28, 1.44, 1.60, 1.76, and 1.92 mg GAE/mL, respectively. Other treatments were used, as shown in Section 3.4.2. The adsorption capacity of each sample was calculated according to Equation (1).

To further investigate the adsorption properties of the resin, Langmuir (Equation (4)) and Freundlich (Equation (5)) models were used to analyze the data.

(4) $$\mathrm{Langmuir}:\;\frac{C_{e}}{q_{e}}=\frac{1}{K_{L}q_{m}}+\frac{C_{e}}{q_{m}}$$

(5) $$\mathrm{Freundlich}:\;lnq_{e}=\frac{1}{n}lnC_{e}+lnK_{F}$$

q_e_ is the adsorption equilibrium capacity (mg). C_e_ is the TPC of the solution after the adsorption equilibrium (mg/mL). qm is the maximum theoretical adsorption capacity (mg). K_L_ is the Langmuir constant. K_F_ is the theoretical saturation adsorption capacity. n is the adsorption driving force.

#### 3.4.5. Adsorption Kinetics

The mixtures of the extraction solution and resin were shaken for 0, 5, 10, 15, 30, 60, 90, 120, and 180 min, respectively. Other treatments were used, as shown in Section 3.4.2. Their adsorption kinetic properties were analyzed using pseudo-first-order (Equation (6)) and pseudo-second-order (Equation (7)) models.

(6) $$ln(q_{t}-q_{e})=-k_{1}t+lnq_{e}$$

(7) $$\frac{t}{q_{t}}=\frac{1}{k_{2}q_{e}^{2}}+\frac{t}{q_{e}}$$

q_e_ is the adsorption equilibrium capacity (mg). q_t_ is the adsorption capacity (mg) at interval time t. k_1_ and k_2_ are the rate constants of pseudo-first-order kinetic and pseudo-second-order kinetic models, respectively.

#### 3.4.6. Desorption Solution

Adsorption saturated macroporous resins were desorbed with 0%, 20%, 40%, 60%, 80%, and 100% ethanol solutions, respectively. The solutions were shaken at 25 °C and 150 rpm for 12 h. The desorption capacity was calculated according to Equation (2).

#### 3.4.7. Dynamic Adsorption and Desorption

A glass column with an inner diameter of 1.5 cm and a length of 60 cm was selected for dynamic adsorption and desorption experiments. The experiment was carried out by filling the macroporous resin with a height of 9 cm (diameter/height ratio 1:6) (Figure 13).

Dynamic adsorption: RLE solution was added to the column at flow rates of 2, 3, and 4 BV/h, respectively. The effluent of each column volume was collected and TPC values were determined.

Dynamic desorption: The adsorption column was washed with deionized water until the effluent was colorless, and the optimal desorption solution was added at flow rates of 4, 5, and 6 BV/h, respectively. The effluents were collected every 0.5 BV (bed volume) and their TPC was determined.

### 3.5. HPLC–MS Analysis

The extract solution (1 mL) was filtered through a 0.22 μm membrane and stored at 4 °C until HPLC–MS analysis. The solutions were analyzed using a Thermo Scientific QE Orbitrap mass spectrometry system (Thermo Fisher Scientific Inc., Waltham, MA, USA) equipped with an electrospray interface. The column was Hypersil GOLD (C18 column, 100 mm × 2.1 mm, 3 μm). The sample injection volume was 1 µL, and the gradient process was divided into phase A (water + 0.1% formic acid) and phase B (acetonitrile). The chromatography and mass spectrometry conditions were set according to Yang et al. [40]. 

### 3.6. Antioxidant Activity

The scavenging activity of DPPH/ABTS was identified according to the experimental method of Yang et al. [40] and calculated using Equation (8):

(8) $$\mathrm{Scavenging}\;\mathrm{rate}\;(\%)=1-\frac{A_{1}-A_{0}}{A}\times \;100\%$$

A_1_ is the sample + DPPH/ABTS. A_0_ is the sample. A is the DPPH/ABTS solution.

### 3.7. Anti-Enzyme Activity

α-Amylase and α-glucosidase inhibition activities were determined according to Li et al. [15] and calculated according to Equation (9).

(9) $$\mathrm{Inhibition}\;\mathrm{rate}\;(\%)=(1-\frac{C-D}{A-B})\times 100\%$$

A is the PBS + enzyme. B is the PBS. C is the PBS + enzyme + sample. D is the PBS + sample.

### 3.8. Statistical Analysis

Each experiment was repeated at least three times, and the experimental data were expressed as the mean ± standard deviation. The RSE data were analyzed using Design-Expert 11 software. Analysis of variance (ANOVA) was performed using IBM SPSS statistics 22.0 software. HPLC–MS analysis was performed using Xcalibur software 3.0 (Thermo Fisher Scientific Inc., Waltham, MA, USA).

## 4. Conclusions

Based on the single-factor and RSE results, the optimum extraction process was obtained by combining the three extraction methods of US, HG, and SE. The results showed that SE pre-treatment effectively destroyed the internal structure of the plant materials, so that the subsequent US and HG could better release the phenolic compounds. SE had a double-edged effect, which was more conducive to the release of active substances as a pre-treatment method. The TPC of RLE through SE/HG/US-assisted extraction reached 136.30 mg/g~140.51 mg/g. After single or double XDA-6 purification, the TPCs of RLP-1 and RLP-2 were 738.98 mg/g and 905.27 mg/g, respectively, and the contents of typical phenolic compounds were also increased. Finally, RLP-2 showed the highest antioxidant activity, and α-glucosidase and α-amylase inhibition activities, and its Q3GR content reached 199.69 mg/g. 

## Figures and Tables

**Table 1:** The results of RSE.

No.	Factor			TPC (mg/g)
	A (Time, min)	B (Temperature, °C)	C (Liquid–Solid Ratio, mL/g)	
1	5	105	24	81.47
2	25	105	24	98.90
3	5	125	24	93.54
4	25	125	24	93.54
5	5	115	8	80.20
6	25	115	8	87.58
7	5	115	40	90.82
8	25	115	40	103.58
9	15	105	8	70.50
10	15	125	8	80.35
11	15	105	40	93.50
12	15	125	40	93.16
13	15	115	24	112.12
14	15	115	24	115.75
15	15	115	24	111.77
16	15	115	24	113.67
17	15	115	24	115.13

**Table 2:** ANOVA for RSE.

	Sum of Squares	Df	Mean Square	F-Value	*p*-Value	Significant
Model	3096.85	9	344.09	96.67	<0.0001	**
A—Time	176.44	1	176.44	49.57	0.0002	**
B—Temperature	32.89	1	32.89	9.24	0.0189	*
C—Liquid–solid ratio	487.19	1	487.19	136.87	<0.0001	**
AB	75.95	1	75.95	21.34	0.0024	**
AC	7.24	1	7.24	2.03	0.1970	ns
BC	25.96	1	25.96	7.29	0.0306	*
A^2^	258.04	1	258.04	72.49	<0.0001	**
B^2^	824.78	1	824.78	231.71	<0.0001	**
C^2^	987.37	1	987.37	277.39	<0.0001	**
Residual	24.92	7	3.56			
Lack of Fit	12.46	3	4.15	1.33	0.3815	ns
Pure Error	12.46	4	3.12			
Cor Total	3121.77	16				

*: Significant difference at *p* < 0.05; **:significant difference at *p* < 0.01; ns: non-significant difference at *p* ≥ 0.05.

**Table 3:** Combined processes.

No	Name	1st Step	2nd Step	3rd Step	TPC mg/g
1	HUSE	HG	US	SE	126.23 ± 2.09 b
2	HSEU	HG	SE	US	120.16 ± 9.63 b
3	SEHU	SE	HG	US	136.30 ± 6.08 a
4	SEUH	SE	US	HG	140.51 ± 1.67 a
5	USEH	US	SE	HG	121.48 ± 2.77 b
6	UHSE	US	HG	SE	125.54 ± 2.23 b
7	US	US			116.51 ± 7.96 c
8	HG	HG			101.96 ± 9.28 c
9	SE	SE			113.86 ± 5.31 c

SE: Deionized water, 115·°C, 15 min, 29 mL/g. HU: 60% ethanol, 4000 r/min, 1 min, 40 mL/g. US: 60% ethanol, 40 mL/g, 120 W, 30 min. Different lowercase letters (a–c) show significant difference (*p* < 0.05).

**Table 4:** Adsorption isotherm equation and parameters.

Model	Equations	Parameters		
Langmuir	Ce/q_e_ = 0.0535 + 0.0193Ce	K_L_ (mL/mg)	q_m_ (mg/g)	R^2^
		0.36	51.93	0.9872
Freundlich	Lnqe = 0.7368lnCe + 1.9059	K_F_ [mg/g (L/mg)^1/n^]	1/n	R^2^
		13.29	0.74	0.9721

**Table 5:** Adsorption kinetic equations and parameters.

Model	Equations	Parameters		
Pseudo-first-order	ln(q_e_ − q_t_) = −0.0330t + 3.3719	K_1_	q_e_	R^2^
		0.0760	29.13	0.9460
Pseudo-second-order	t/qt = 0.02283t + 0.24792	K_2_	q_e_	R^2^
		0.0021	43.80	0.9988

**Table 6:** IC_50_ values of DPPH and ABTS methods (μg/mL).

Extract	DPPH	ABTS
RLE	67.71 ± 1.38	14.72 ± 0.37
RLP-1	33.99 ± 0.35	7.81 ± 0.14
RLP-2	20.63 ± 1.09	5.98 ± 0.18
Vc	11.55 ± 1.12	7.49 ± 0.27

**Table 7:** Single-factor experimental design.

Factor	Level				
Temperature (°C)	105	110	115	120	125
Time (min)	5	10	15	20	25
Liquid-solid ratio (mL/g)	8	16	24	32	40

**Table 8:** RSE design.

Factor	Level		
	−1	0	1
Temperature (°C)	105	115	125
Time (min)	5	15	25
Liquid-solid ratio (mL/g)	8	24	40

**Table 9:** Properties of six macroporous resins.

Resin Type	Polarity	Surface Area (m^2^/g)	Particle Size (0.35–1.25 mm) (%)
LX-83	Nonpolar	876.19	98.20
LX-12	Weak polar	545.09	99.00
XDA-6	Middle polar	690.89	97.60
D101	Nonpolar	768.31	96.80
D101C	Nonpolar	677.01	97.00
AB-8	Weak polar	568.66	96.20

## Data Availability

Not applicable.

## Supplementary Materials

The following supporting information can be downloaded at: https://www.mdpi.com/article/10.3390/molecules28176321/s1, Figure S1. Changes of three extract TPC under different SE treatment times; Figure S2. Changes in phenolic compounds before and after SE treatment.
