# Supplementary material for: An Integrated Extraction–Purification Process for Raspberry Leaf Polyphenols and Their In Vitro Activities
Source: Molecules. 2023 Aug 29;28(17):6321. doi: 10.3390/molecules28176321 (PMC10489654; doi:10.3390/molecules28176321)
Supplement: Supplementary file 1 [file molecules-28-06321-s001.zip › molecules-2559973-supplementary.pdf]

Supplementary materials

1 Effect of SE on samples

RLE, RLP-1 and RLP-2 were treated at 115℃ for 10, 20, 30, 40 and 50 min, respectively. Their TPC, typical phenolic compounds, antioxidant and anti-enzyme activities were determined and analyzed.

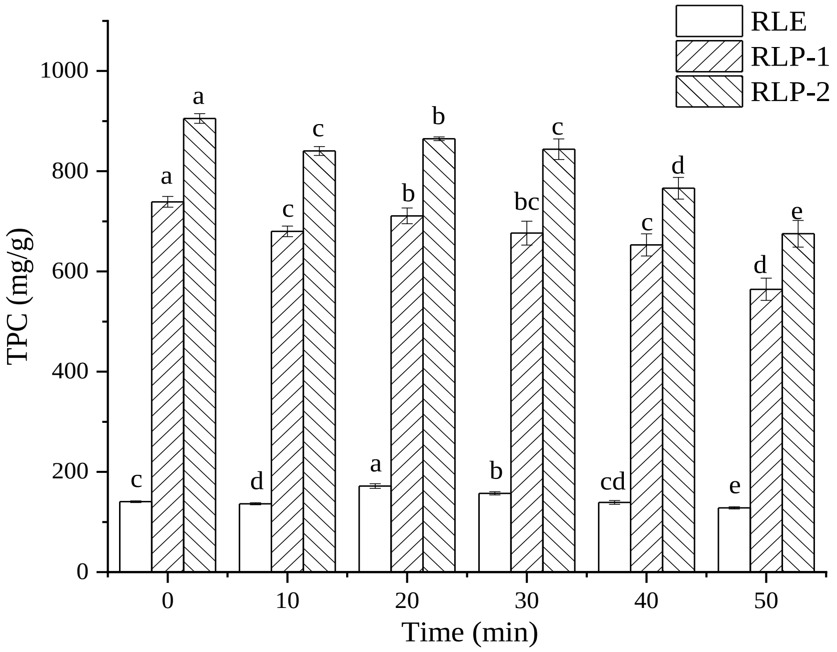

Figure S1. Changes of three extract TPCs under different SE treatment times.

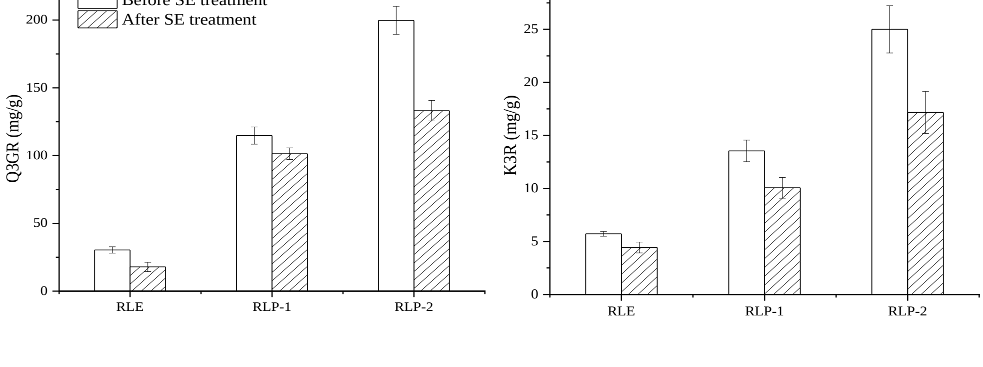

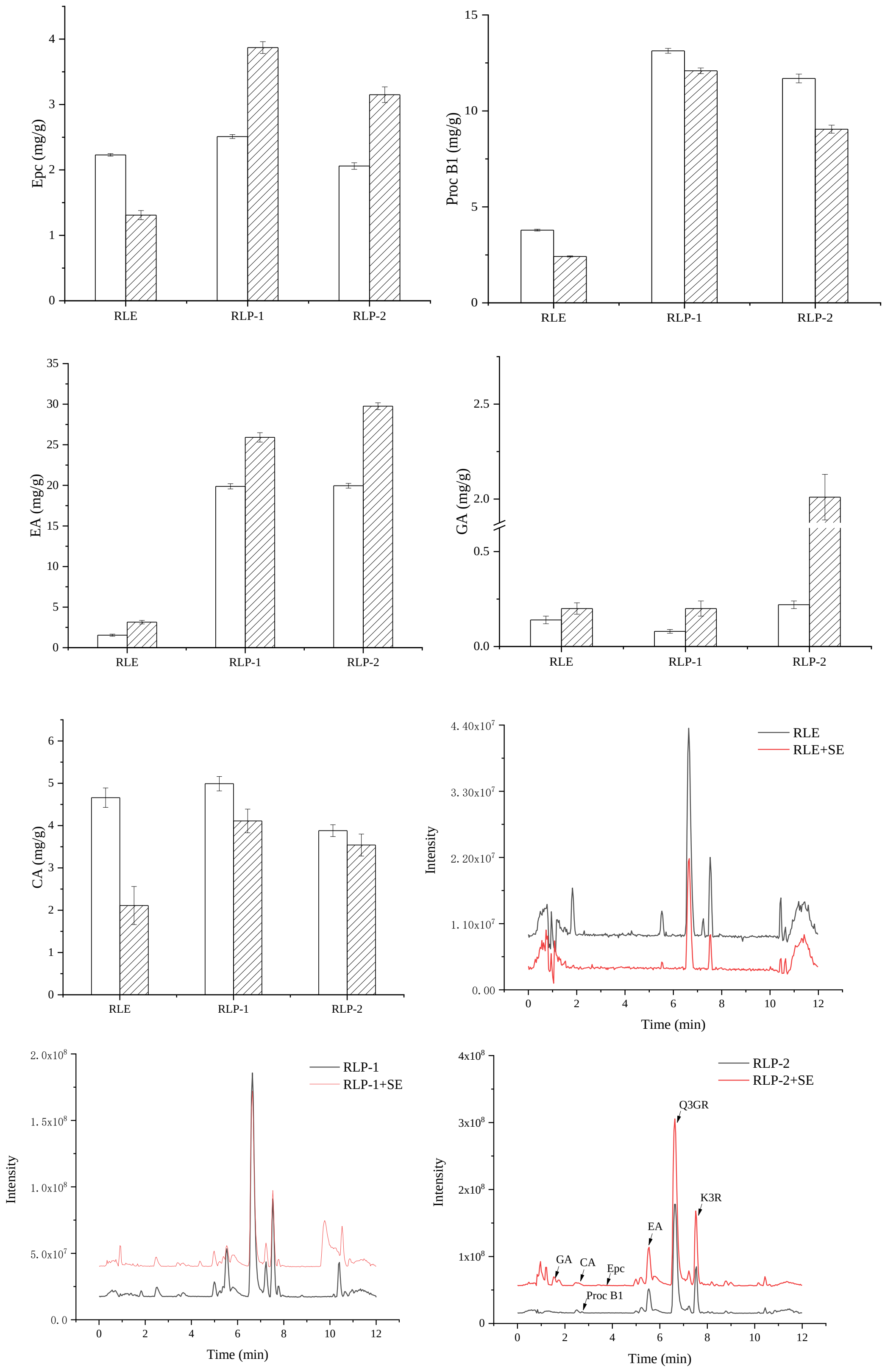

Figure S2. Changes of phenolic compounds in samples before and after SE treatment.
